# Transforming Dental Education: Artificial Intelligence Simulation to Bridge Patient‐Provider Communication Gaps

**DOI:** 10.1002/jdd.13960

**Published:** 2025-06-20

**Authors:** Salma Elwazeer

**Affiliations:** ^1^ Woody L. Hunt School of Dental Medicine Texas Tech University Health Sciences Center El Paso El Paso Texas USA

## 1 Problem

Effective communication is critical in dental education, and all dental students should show competence in patient communication, especially when addressing sensitive topics like substance use disorders (SUD) [1]. While students are introduced to Motivational Interviewing (MI) and the Screening, Brief Intervention, and Referral to Treatment (SBIRT) framework, many struggle to translate this theoretical knowledge into practice [2]. Traditional methods such as classroom instruction, role‐playing, and faculty‐led demonstrations provide foundational expertise but often lack the personalized feedback, consistency, and adaptability needed for skill mastery due to time constraints [1]. Without adequate and interactive training, students may lack confidence in navigating complex patient conversations, leading to suboptimal outcomes and patient‐provider mistrust [1]. Thus, there is a pressing need for an innovative, scalable, and interactive training approach to bridge the gap between learning MI/SBIRT techniques and applying them effectively in clinical settings.

## 2 Solution

**FIGURE 1:**
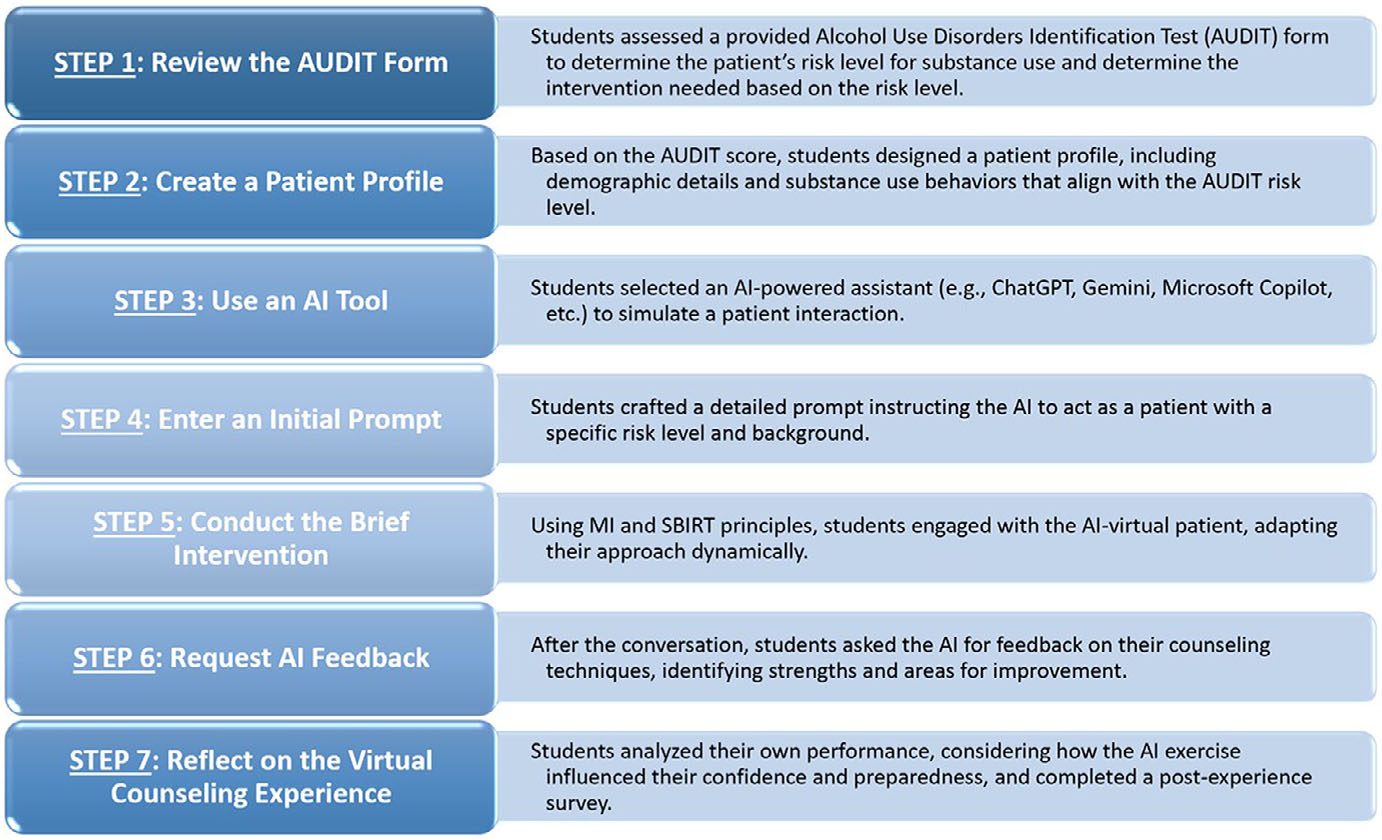
Artificial intelligence (AI) screening, brief intervention, and referral to treatment (SBIRT) assignment workflow.

**FIGURE 2:**
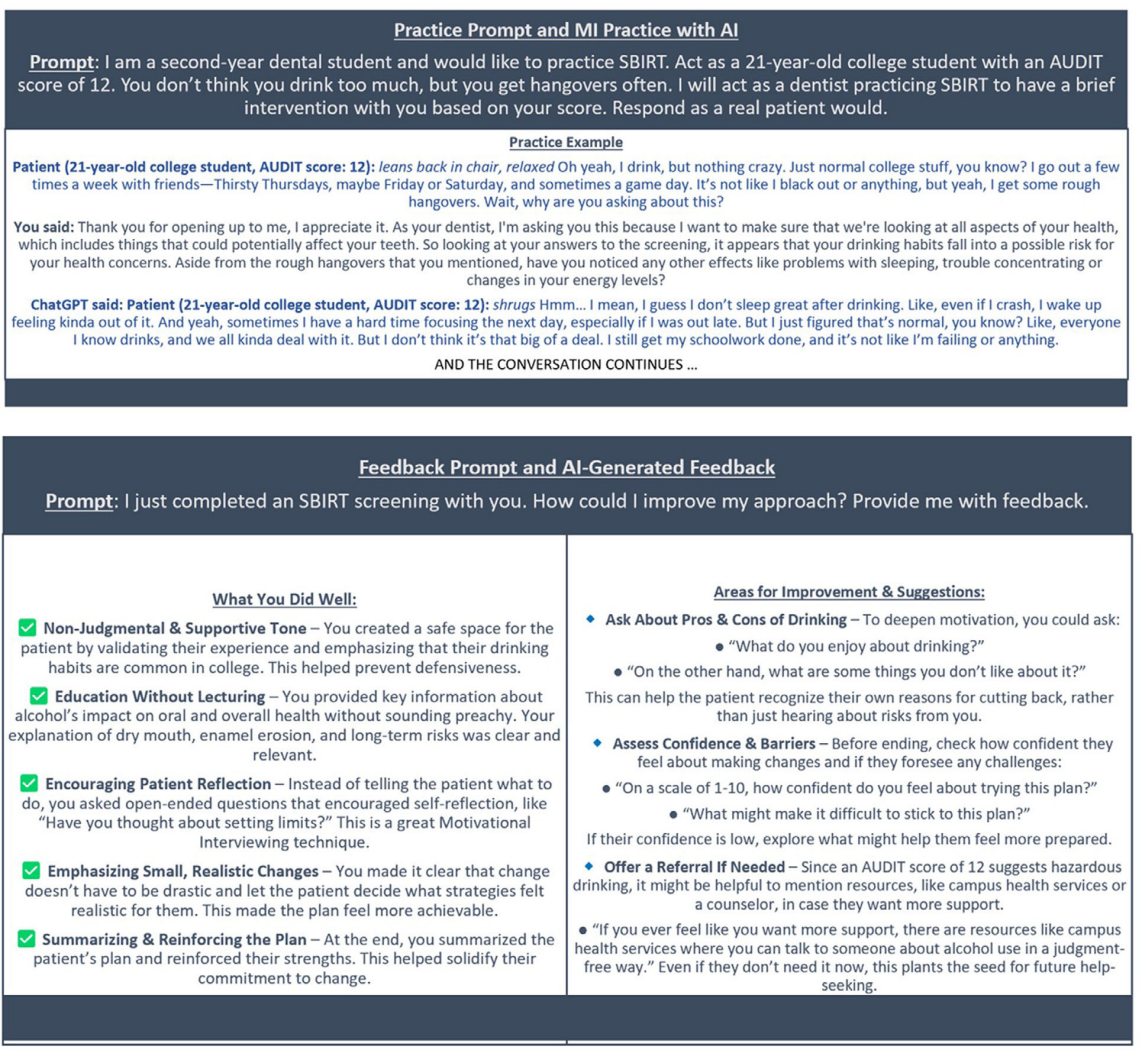
Example of artificial intelligence (AI)‐facilitated motivational interviewing (MI) encounter and AI‐generated feedback.

Artificial intelligence (AI) is increasingly transforming healthcare education by offering scalable, interactive learning opportunities [3, 4]. To support this innovation, an AI‐facilitated SBIRT assignment was implemented at the Woody L. Hunt School of Dental Medicine (WLHSDM) as a pre‐objective structured clinical examination (pre‐OSCE) formative training method. This assignment allowed students to engage with AI‐driven virtual patients through text‐based simulations, providing a low‐risk, yet a high‐impact environment to refine their communication skills. Students followed a step‐by‐step process, including selecting an AI tool of their choice, writing prompts, conducting a simulated intervention, and requesting feedback from the AI. An overview of the assignment structure was shared with the students and is presented in Figure 1. A sample student AI‐facilitated patient‐provider MI communication and the AI‐driven personalized feedback are shown in Figure 2.

## 3 Results

**FIGURE 3:**
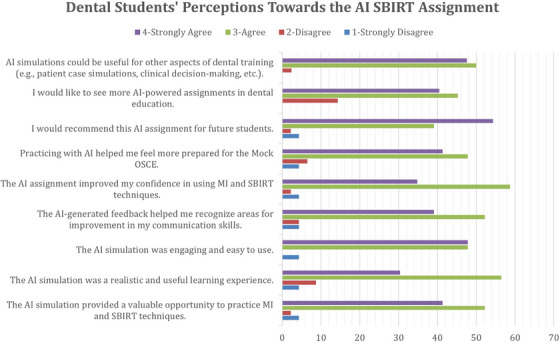
Dental students’ perceptions of the artificial intelligence (AI) screening, brief intervention, and referral to treatment (SBIRT) assignment.

A total of 47 out of 60 dental students completed the post‐assignment evaluation survey (78% response rate). All respondents chose to use ChatGPT (version 3.5 or 4.0) to complete the simulation. Overall, students responded positively to the experience. Around 93% agreed or strongly agreed that the AI simulation provided a valuable opportunity to practice MI and SBIRT techniques. Similarly, nearly 96% found the simulation engaging and easy to use. Feedback from the AI assistant was also seen as beneficial, with over 91% agreeing that it helped them recognize areas for improvement in their communication skills. In terms of skill development, approximately 93% of students agreed or strongly agreed that the AI assignment improved their confidence in using MI and SBIRT techniques, and over 90% felt more prepared for the Mock OSCE as a result of the exercise. Interest in the continued use of AI in education was also high, with 86% expressing a desire to see more AI‐facilitated assignments integrated into dental education, and nearly 98% believed AI simulations could be beneficial for other aspects of dental training, such as patient case simulations and clinical decision‐making. Figure 3 summarizes dental students’ perceptions of the AI‐facilitated SBIRT Assignment.

These findings suggest that the AI‐powered SBIRT assignment was not only effective in building students' communication skills and confidence but was also well‐received as a scalable, innovative tool for enhancing dental education.

## Conflicts of Interest

The authors declare no conflicts of interest.
